# Electrical Stimulation and Conductive Polymers as a Powerful Toolbox for Tailoring Cell Behaviour *in vitro*

**DOI:** 10.3389/fmedt.2021.670274

**Published:** 2021-07-29

**Authors:** Igor Rocha, Gabrielle Cerqueira, Felipe Varella Penteado, Susana I. Córdoba de Torresi

**Affiliations:** Instituto de Química, Universidade de São Paulo, São Paulo, Brazil

**Keywords:** electrical stimulation, conductive polymers, biomaterials, cell culture, cells-material interactions

## Abstract

Electrical stimulation (ES) is a well-known method for guiding the behaviour of nerve cells in *in vitro* systems based on the response of these cells to an electric field. From this perspective, understanding how the electrochemical stimulus can be tuned for the design of a desired cell response is of great importance. Most biomedical studies propose the application of an electrical potential to cell culture arrays while examining the cell response regarding viability, morphology, and gene expression. Conversely, various studies failed to evaluate how the fine physicochemical properties of the materials used for cell culture influence the observed behaviours. Among the various materials used for culturing cells under ES, conductive polymers (CPs) are widely used either in pristine form or in addition to other polymers. CPs themselves do not possess the optimal surface for cell compatibility because of their hydrophobic nature, which leads to poor protein adhesion and, hence, poor bioactivity. Therefore, understanding how to tailor the chemical properties on the material surface will determine the obtention of improved ES platforms. Moreover, the structure of the material, either in a thin film or in porous electrospun scaffolds, also affects the biochemical response and needs to be considered. In this review, we examine how materials based on CPs influence cell behaviour under ES, and we compile the various ES setups and physicochemical properties that affect cell behaviour. This review concerns the culture of various cell types, such as neurons, fibroblasts, osteoblasts, and Schwann cells, and it also covers studies on stem cells prone to ES. To understand the mechanistic behaviour of these devices, we also examine studies presenting a more detailed biomolecular level of interaction. This review aims to guide the design of future ES setups regarding the influence of material properties and electrochemical conditions on the behaviour of *in vitro* cell studies.

## Introduction

Since the discovery of conductive polymers (CPs) in the late 1970's, interest in these materials has steadily grown (1). Currently, there are a wide variety of new polymers and their derivatives, consisting basically of aromatic rings or linear chains with alternating single and double bonds, which allow the transport of electrons through conjugated π orbitals. These materials combine the electrical and optical properties of semiconductors with the mechanical and processing properties of polymers. Table 1 shows the structures of the most widely used CPs, which are applied in the development of rechargeable batteries, electrochromic devices, conductive plastics (2), drug-delivery devices (3), light-emitting diodes (4), sensors and biosensors (5, 6), electrocatalysis, and corrosion protection (7–9).

**Table 1:** Schematic structures of the main conductive polymers.

**Name**	**Linear structure**
Polyacetylene	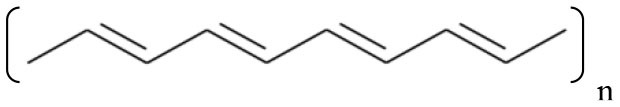
Polypyrrole (PPy)	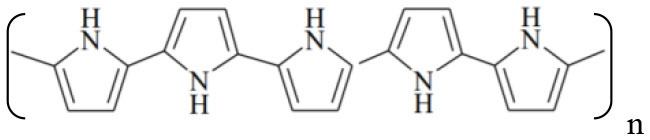
Polyaniline (PANI)	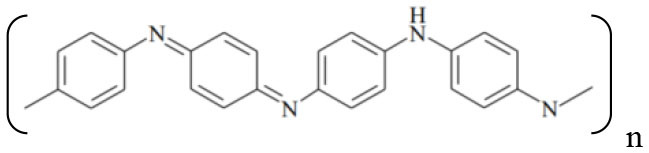
Poly(3,4-ethylenedyoxythiopene) (PEDOT)	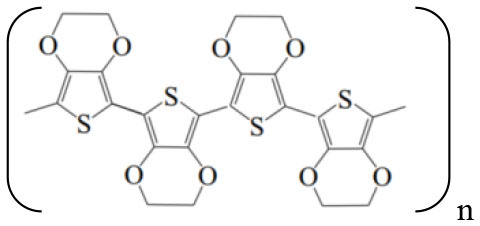

CPs generally present band gap (E_g_) values higher than 1.5 eV, which makes them insulating materials. From oxidation or reduction of polymeric chains, decrease in E_g_ then happens, allowing these materials to become conductors. These processes occur with the formation of polarons, radical ions associated with distortion of the polymeric chain, and bipolarons, namely, pairs of similar charges associated with strong local distortions of the polymeric network (10). The value of E_g_ can be modulated by different functional groups. The substituent also influences properties, such as solubility, thermal stability, oxidation potential, electronic structure, and conductivity of the polymer (11).

PEDOT, for example, presents high conductivity and can be found in both neutral (reduced) and doped (oxidised) forms, with interconversion between forms taking place through primary and secondary doping processes. In general, the primary doping process of CPs refers to the addition of substances that decrease E_g_, changing the electronic properties of the polymeric structure and enabling the electron conduction property (12). Secondary doping, according to MacDiarmid and Epstein (13), is the insertion of an apparently inert substance that provides an additional increase in the conductivity of a primarily doped polymer. Secondary doping differs from primary doping in that new enhanced properties can persist even with the complete removal of secondary doping. Doping reactions of the conjugated polymers are related to the transport of ions and electrons in the material. Depending on the types of charge compensation used during polymerisation, both insertion and release of cations and anions can occur during the electrochemical redox process. If a polyanion is used as a supporting electrolyte during the preparation of the CP, that polyanion is then immobile during redox processes, since it is incorporated into the polymer matrix on a molecular scale. Thus, two possible reactions take place during the doping/de-doping processes, as outlined in Equations 1, 2:


(1) $${\text{PEDOT}}^{0}{\text{A}}^{-}{\text{M}}^{+}⇌{\text{PEDOT}}^{+}{\text{A}}^{-}+{\text{e}}^{-}$$

(2) $${\text{PEDOT}}^{0}⇌{\text{PEDOT}}^{+}{\text{X}}^{-}+{\text{e}}^{-}$$

where A is the polyanion used during synthesis, and M^+^ and X^−^ are the cation and anion of the salt used as a supporting electrolyte during electrochemical characterisation.

It has been reported that CPs, such as polypyrrole (PPy) and PEDOT doped with polysaccharides, namely, hyaluronic acid and heparin, can potentially be used in living tissue engineering as material for neural electrodes (14–16), because they can serve as biocompatible and biodegradable electroactive materials to increase the regeneration of peripheral nerves and other tissues (17). Lu et al. (18) and Yuk et al. (19) presented a way to produce a hydrogel made only of a mixture of PEDOT and polystyrene sulphonate (PEDOT:PSS) that could be 3D-printed, allowing the fabrication of devices with controllable shapes, which is important for tissue engineering applications. The authors printed a soft neural probe that could monitor the neural activities of a living mouse.

Among CPs, PPy is an intrinsic conductive polymer that has received considerable attention because of its high electrical conductivity and electrochemical stability (20). It is well-known in the literature that PPy can be found in different oxidising states. The degree of PPy doping leads to more or less conductive species, which are usually reversible, depending only on the amount of oxidant used or the potential applied to an electrode (21, 22). Studies have shown that it is able to support the *in vitro* growth of various types of cells (23). *In vivo* studies have also confirmed that PPy is non-cytotoxic to some extent, improving the interaction of nerve cell cultures to stimulate peripheral nerve regeneration (24). Knowing the concentration at which the polymer does not present cytotoxicity is fundamental for its applicability, especially in the biomedical area, to prevent toxicity and damage to the organism.

PANI is another widely used CP because of its highly conductive properties and its availability in different oxidation states, which can range from fully oxidised to fully reduced and are affected by pH. The most investigated form of PANI is the emeraldine base (PANI-EB), given its stability at room temperature and its transformation to the emeraldine salt form (PANI-ES) after doping with acid (protonated), a form that is known to be highly conductive (25).

It is worth mentioning that the development of biotechnological applications based on CPs usually includes the formation of blends and composites. Therefore, understanding the interactions among the components in the material is crucial for controlling the *in vitro* behaviour. Alemán et al. (26) assessed the interaction between CPs and DNA sequences, with major implications in numerous medical applications, from diagnosis to gene therapy. The interaction of post-doped electroactive materials with DNA was traditionally attributed to the tendency of DNA to interact with positively charged molecules. However, it has been found that some conductive polymers, such as PPy, are able to interact by forming specific interactions with well-defined nucleotide sequences of plasmid DNA. This selectivity suggests that the polymer-DNA hybrids are stabilised not only by electrostatic interactions but also by specific interactions depending on the chemical environment, spatial disposition, and orientation of the chemical groups.

Various studies have made use of the electrical nature of CPs to change their properties by applying ES (27–29). These procedures can also affect living structures attached to these materials by altering their chemical nature. During the eighteenth century, Luigi Galvani started research on frogs, analysing the effect of an electric field on the muscle-nerve system of the animal, which resulted in contractions. He concluded that there was some inherent electricity in the animal that could allow an external stimulus to flow in the body. For this study, Galvani became an important name in electrophysiology science (30, 31). Centuries later, scientists still studied the effect of the electric field in living organisms, namely, Patel and Poo (32) analysed the reaction of *Xenopus* neurons after applying a 1–10 V cm^−1^ steady state electric field. They observed that the cells changed the direction of neurite growth with the direction of the electric field and that the neurites had enhanced growth in the cathode direction. Their results indicated that the electric field not only had a biological effect, as noticed by Galvani but also could promote cell growth and organisation and could alter molecule migration in the neuronal membrane surface.

Moreover, the muscles and the neurosystems are not the only types of cells that can suffer an electric field effect. Fukada and Yasuda (33) and Anderson and Eriksson (34) investigated the piezoelectric properties of bone cells, pointing out that piezoelectricity enabled the use of an electric field in these cells. Valentini et al. (35) used polyvinylidene fluoride (PVDF), a piezoelectric polymer, to enhance neurite outgrowth of Nb2a cells with charged polymers, indicating that the materials could also assist ES.

From this perspective, Wong et al. (36) showed that PPy could be used as a biomaterial for cell culture of aortic endothelial cells, and they also noticed that the polymer charge affected the cell shape, indicating that the charge on the surface could regulate cell growth but did not affect the cell viability.

A very recent study by da Silva et al. (37) presented a study concerning the effect of ES on protein and cell adhesion over a copolymer of PEDOT and poly(d,l-lactic acid) (PEDOT-co-PDLLA) film. They investigated the effect of a + 0.5 V and a −0.125 V ES in a film of the copolymer over a gold quartz electrode, monitoring the adsorption of fibronectin and the adhesion of NIH-3T3 with an electrochemical quartz crystal microbalance with dissipation (QCM-D). They noticed that ES increased fibronectin adsorption on the PEDOT-co-PDLLA surface and that the stimuli also promoted an improvement of fibroblast adhesion over the film and increased cell proliferation.

Previous reviews have focused on the effect of ES on cell behaviour and closely examined the cell response regarding viability, morphology, and gene expression. In this study, we attempt to summarise these interactions mediated mainly by CPs, and we look more closely at the material synthesis, properties, and electrochemical setup (38–63).

## Review

### Polypyrrole-Based Materials

**Figure 1:**
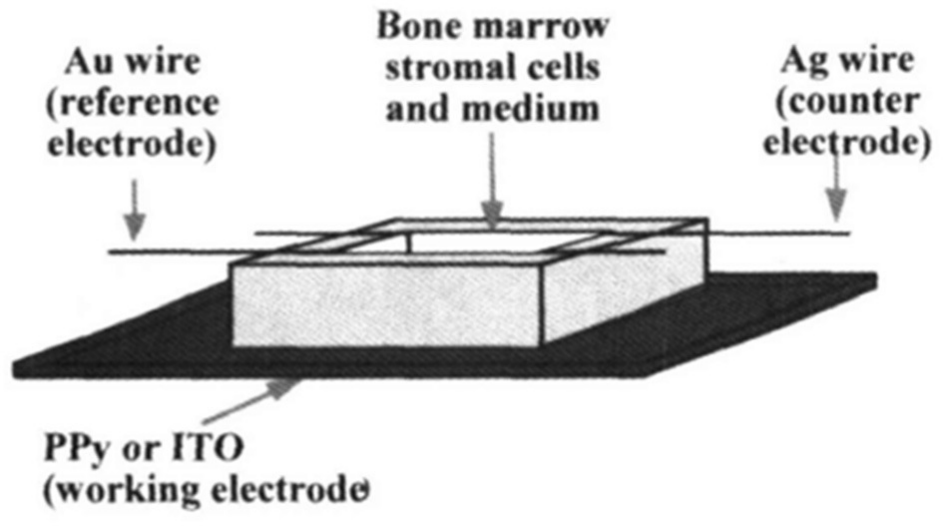
Scheme for Electrical stimulation (ES) of BMSC on polypyrrole (PPy) thin films. Reproduced from (64) with the permission of the Materials Research Society.

Shastri et al. (64) presented an ES study using PPy as a conductive substrate able to generate a response in the differentiation of bovine bone marrow stroma cells (BMSCs). They chose this CP for this cell type based on a study by Yasuda (33) investigating the piezoelectric behaviour of bone tissue. The electrode is based on electropolymerised PPy over an indium tin oxide (ITO) substrate. For BMSC growth, they used osteogenic supplements, ascorbic acid, dexamethasone, and β-glycerophosphate to enhance the capability of osteogenic differentiation. The ES setup is based on the use of a PPy electrode as the anode, a gold wire acting as a cathode, and the quasi-reference electrode as the silver wire (Figure 1). The authors applied a steady field of 20 V m^−1^ for 1 h, showing cell viability over the PPy substrate similar to the control (tissue culture polystyrene, TCPS) and better than that on the ITO without the PPy film. The analysis of alkaline phosphate (ALP) activity (an important marker to indicate osteogenic differentiation) showed that cells cultured over the PPy film resulted in higher levels of ALP activity, which was enhanced after ES only on the PPy film. Therefore, these results were presented as an effect of the surface chemistry of PPy films, with the negatively charged surface being important for the adhesion of positively charged proteins of cells, such as fibronectin, which acts in favour of cell adhesion and osteogenic differentiation.

**Figure 2:**
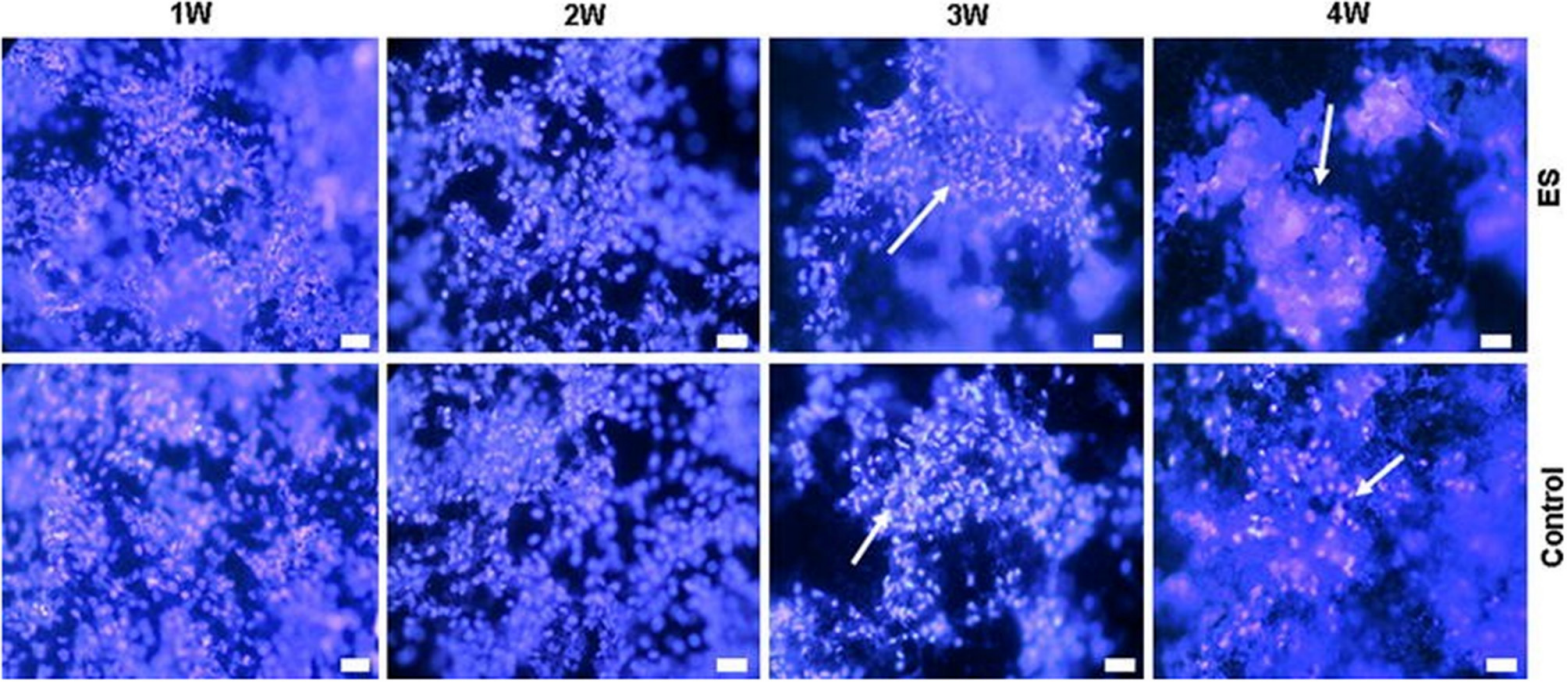
Electrical stimulation study on Saos-2 cells on PLLA/PPy/HE membranes. The arrowheads show the formation of nodules in the highest periods (Hoechst staining, bar 10 μm). Adapted from (65) with permission from the publisher.

Another study related to the ES of bone cells was published by Meng et al. (65). In that study, the authors used osteoblast-like Saos-2 cells, which are important for the regeneration of bone tissue, and a PPy/polylactide (PLLA)/heparin (HE) composite was synthesised *via* chemical polymerisation. The reaction yielded a solution that was cast onto a Teflon plate forming a membrane. Cell culture and growth were expected to yield bone nodule mineralisation and to form inorganic elements, mainly calcium and phosphate. These minerals are present in hydroxyapatite, the main structural mineral in bone composition. After incubation in a standard medium for 2 days, the cells were cultured for 24 h in wells in the same standard medium, which was replaced with a mineralisation medium, and 200 V mm^−1^ ES was applied for three periods of 6 h for 6 days. The cultures were then left to grow for 3 more weeks without any ES. The control group and the experimental group showed divergences on week 3, when more cell aggregates appeared in the experimental wells than in the control wells. From weeks 3–4, significant growth was observed in the amount of those cell aggregates in the experimental wells (Figure 2).

Scanning electron microscopy (SEM) analysis of mineralisation showed that in both the experimental and control groups, mineral particles appeared in the 1st week and grew over the course of the 4 weeks, as well as osteoblast nodules and aggregations. It is important to highlight that the number and size of mineral particles and the osteoblast nodules/aggregations were much larger in the experimental cultures than in the control cultures. The crystal structure of the mineral obtained by wide-angle x-ray diffraction (WAXD) was remarkably similar to those obtained from natural hydroxyapatite. This finding indicates that the crystallisation process of the experiment resembles the natural process. Moreover, there was a clear augmentation of calcium and phosphate levels when ES was applied.

In a more recent study, Hardy et al. (66) reported a method for creating silk foam-based bone tissue scaffolds, which allows the stimulation of human mesenchymal stem cells (HMSCs) to intensify their osteogenic differentiation. Natural silk proteins and recombinant silk-inspired proteins are well-known base materials for use in tissue scaffold engineering (67–71). The scaffolds were interpenetrated with a self-doped CP composed of pyrrole and 2-hydroxy-5-sulphonic aniline, and polymerisation within the scaffold was performed *in situ* using ammonium persulphate and ferric chloride. The enhanced scaffolds allowed direct ES of the HMSCs residing within them and improved the osteogenic differentiation outcome, which was confirmed by both biochemical assays and qualitative histological analysis. ES also increased the calcium and collagen concentrations, which are important for the formation of the calcified extracellular matrix associated with bone.

In another study with PPy, Richardson et al. (72) presented gold electrodes covered with polypyrrole-p-toluene sulphonic acid (PPy:pTS) with an incorporation of neurotrophin-3 (NT3) on its surface and applied ES to release the incorporated NT3 into a spiral ganglion neuron (SGN) culture. PPy was electrochemically polymerised over a gold electrode, and p-toluene sulphonate (tosylate) was added as the dopant. During the polymerisation process, NT3 was added to the electrochemical cell. For the ES experiments, cells had grown over the electrodes for 24 h, and biphasic current pulses were applied for 1 h (± 1 mA current amplitude, at 250 Hz). After ES, the cells were cultured for 3 days before further analysis. The authors verified that polyornithine, a cell adhesion molecule, improved cell growth when coated over electrodes, and they also showed that boosting the NT3 concentration improved neurite outgrowth. They verified the improvement in cell growth when neurites were cultured close to the electrodes and suggested that the effect of NT3 release combined with ES could explain this behaviour. The systems with ES showed an enhancement of neurite outgrowth in comparison with the one without ES when PPy/pTS/NT3 was used, but when the PPy/pTS electrode was used, ES had no influence. This finding indicated that the release of NT3 had a more important effect on cell growth than ES.

In 2009, Xie et al. (73) presented the preparation of a PPy-based core-shear nanofibre for studies on axon regeneration, aiming to use the material for neural tissue engineering. The material consisted of electrospun fibres made of poly(ε-caprolactone) (PCL) or poly(L-lactide) (PLA) and pyrrole using Fe^3+^ and Cl^−^ as an oxidant and as a dopant, respectively. They noticed that the PPy coating over PCL and PLA presented different morphologies. The material electroactivity was studied by applying a 10-V constant potential for 4 h per day over the random and aligned materials. They calculated the effective current applied as ~250 μA (74, 75). ES produced better results in increasing length when using random nanofibres than with aligned nanofibres, which could be related to the cell growth capability reaching a length limit. The authors analysed the ES effect on cell growth, explaining that the electrical current changed the interaction of the adhesive glycoproteins with the charged PPy chain, improving the adhesion of cells and resulting in a higher growth rate (76). Other possibilities were the change in the electrophoretic distribution resulting in polarisation of the cells, improvement in the rate of protein synthesis, favourable protein conformation, migration of Schwann cells, and formation of an induced field in the cell culture with a molecular and ionic gradient (24).

In another study with PPy, Yan et al. (77) proposed the fabrication of an implant that can be electrically stimulated as a way to treat retinal ganglial cells (RGCs) lesioned by glaucoma disease. For this purpose, the use of a mixed material made of graphene oxide (GO) modified with PPy (PPy-G) is proposed prior to preparing a nanofibrous scaffold by electrospinning a mixture of PPy-G with polylactic-*co*-glycolic acid (PLGA). The electrochemical characterisation led the authors to choose an approach with double-pulsed potential chronoamperometry varying the potential from 0.1 to 1 V cm^−1^, and the reverse potential varied from −0.1 to −1 V cm^−1^ for 1 h day^−1^ for 3 days. The variation of potential was important to establish the best conditions of ES for these cells over the material; and it was observed that at ±1,000 mV cm^−1^, death of RGCs was caused by overpolarisation, so a ±700 mV cm^−1^ step potential was chosen. Applying this ES, the cell length increased compared with that in the non-stimulated group, with a major increase observed in the 6% PPy-G scaffold, which was the most electroactive material.

The authors also verified the ageing of the RGC cultures over the 6% PPy-G material with and without ES. The main viability of this cell type after 10 days was 40%, showing nuclear necrosis and cell apoptosis in the control group. Over the conductive scaffold, the cells without ES presented cell shape alterations, such as a reduction of the round shape, indicating an apoptotic process; however, when the cells were cultured over the scaffold and electrically stimulated, they displayed an increase in cell length, and the morphology was maintained. The authors suggested that ES on the material also promoted an anti-ageing effect on RGCs.

**Figure 3:**
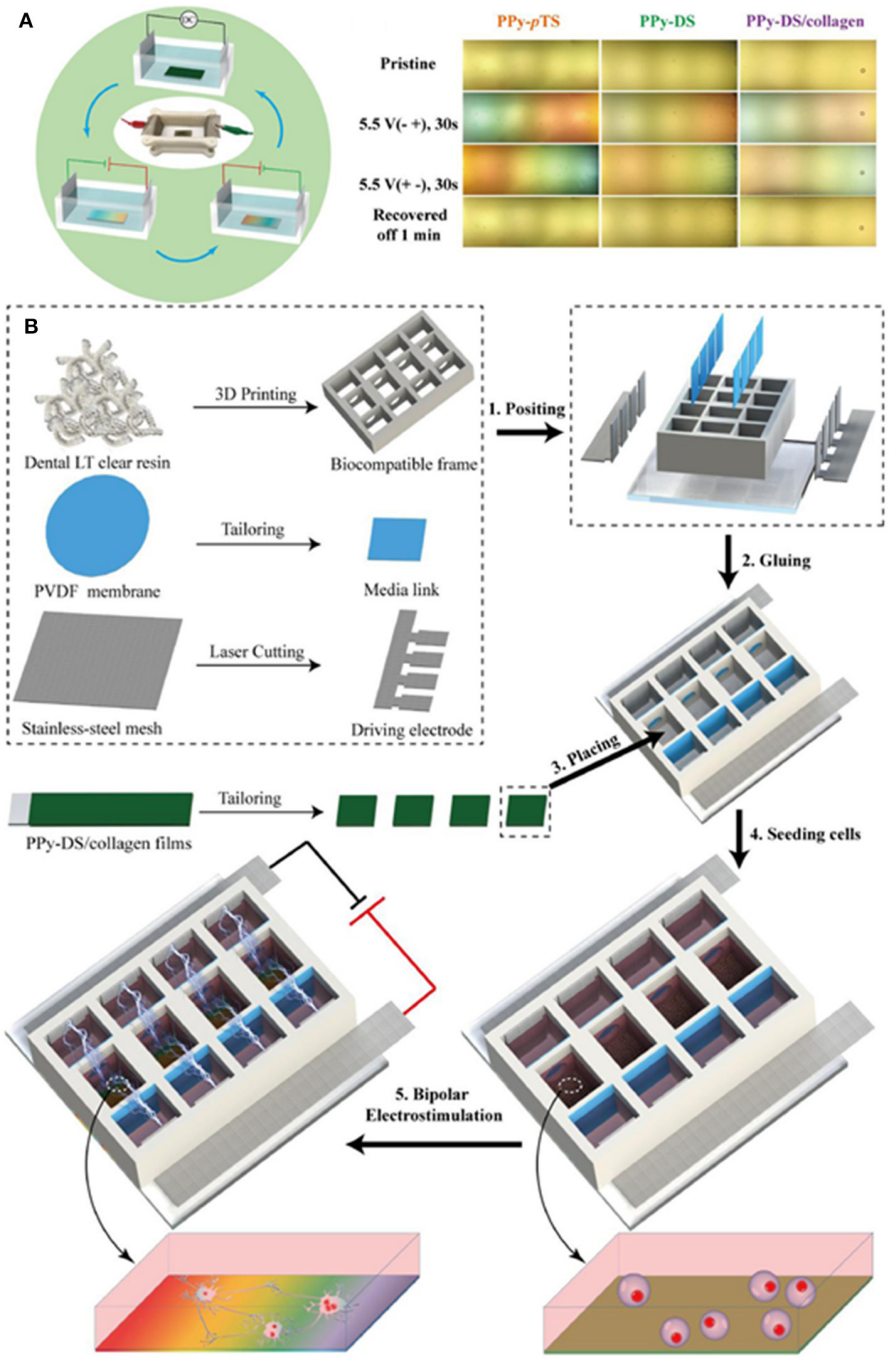
**(A)** Scheme of BPES cycling and CP-immersed bipolar cells. **(B)** Manufacture of BPES platform. Adapted from (78) with permission from the publisher.

A novel approach for applying ES is when the electrode materials are not connected directly to the power supply, but a dipole is created wirelessly between them and the electrodes are connected to the culture medium in which they are immersed. Qin et al. (78) showed that bipolar electrochemistry could offer this effective pathway to modify ES systems into a more desirable contactless mode (Figure 3A). In that study, they presented for the first time the development of a CP-based bipolar electrical stimulation (BPES) system for living cells. PPy films with different dopants were used to demonstrate a reversible and recoverable bipolar electrochemical activity under a low driving DC voltage (<-5.5 V). A BPES prototype enabling wireless and programmable cell stimulation was devised using PPy codoped with dextran sulphate (DS) and collagen (PPy-DS/collagen) as a bipolar electrode and rat pheochromocytoma cells as a model cell line (Figure 3B). Significantly, wireless stimulation enhanced cell proliferation and differentiation. This study established a new paradigm for ES of living cells using CPs as bipolar electrodes, providing an attractive wireless approach to advance the field of medical bionics.

### PANI-Based Materials

**Figure 4:**
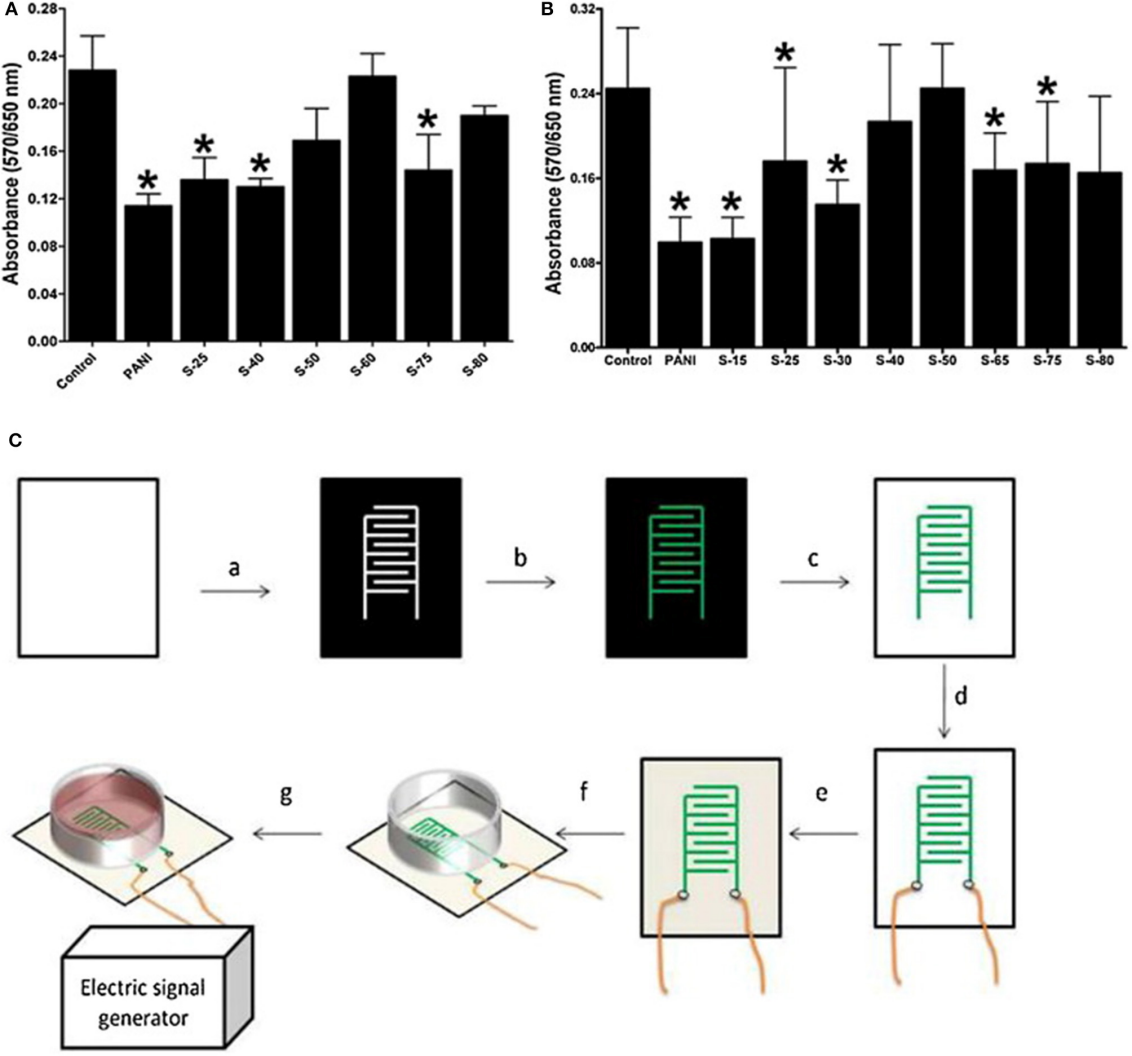
SPAN biocompatibility with **(A)**. BMSCs and **(B)**. MC3T3-E1 cells, without electrical stimulation, after 5 days **(C)**. Fabrication of SPAN-based IDEs: (a) Printing on a PET transparency, (b) layer-by-layer deposition by *in situ* polymerization, (c) toner removal, (d) wire connexion with silver glue, (e) PLA coating, (f) electrode assembly, and (g) power connexion. Adapted from (105) with permission from the publisher. *represents a statistically significant difference in comparison with the control; (1-way RM-ANOVA and Newman–Keuls *post-hoc* analysis with *p* < 0.05, *n* = 6).

Materials based on PANI have also been extensively applied in combination with ES for *in vitro* studies. Min et al. (79) reported a facile fabrication method of self-doped sulphonated polyaniline (SPAN)-based interdigitated electrodes (IDEs) for controlled ES of cancer-type human osteosarcoma (HOS) cells. An increased degree of sulphonation was found to increase the SPAN conductivity, which in turn improved cell attachment and cell growth without ES. However, enhanced cell growth was observed under controlled ES with alternating current at a low applied voltage and frequency (≤800 mV and ≤1 kHz). Cell growth reached a maximum threshold at an applied voltage or frequency, beyond which pronounced cell death was observed. The ES was initially performed with a sine wave generator at a fixed frequency of 1 kHz. The cells were subjected to a steady potential between 0 and 1,600 mV. ES applied through SPAN-based IDEs was found to significantly enhance cell growth, as shown in Figure 4. Furthermore, the growth of the cells increased with the potential and showed no abnormal cellular behaviour or cell death up to 1,000 mV. However, when the applied potential was increased to above 1,200 mV, cell death was more pronounced. A rapid increase in cell growth was observed at a very low applied frequency (≤1 kHz), and it remained constant up to 100 kHz. Nevertheless, significantly enhanced cell death was observed above 200 kHz. The ALP activity profile was similar to the cell proliferation profile, which further confirmed the above results.

**Figure 5:**
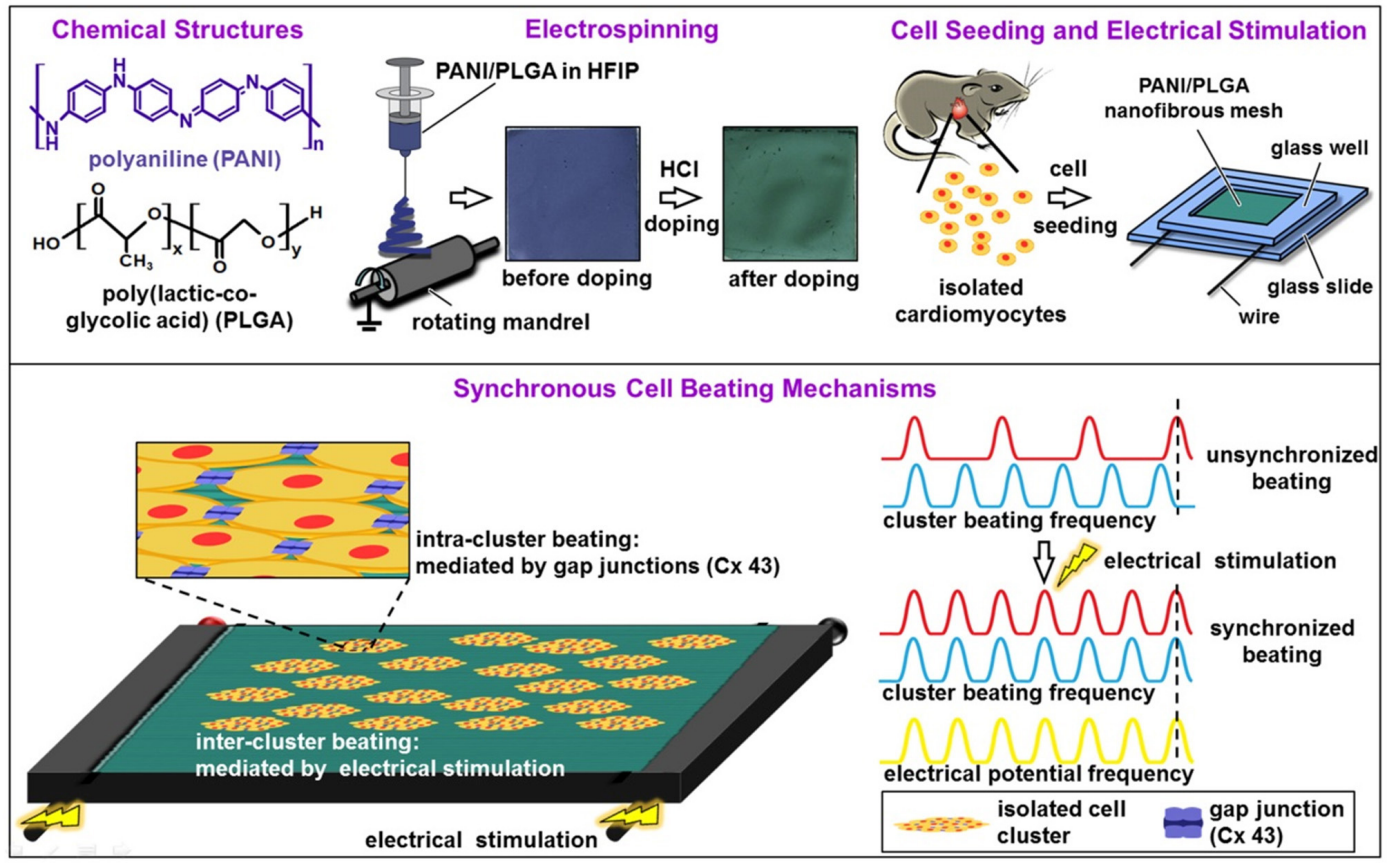
Diagrams showing the fabrication of aligned conductive PANI/PLGA nanofibrous mesh, cell seeding, ES, and the mechanisms involved in the synchronous cell beatings. Adapted from (80) with permission from the publisher.

The study of Hsiao et al. (80) presented the synthesis of a nanofibre mesh of PLGA and PANI for ES of cardiomyocytes. The fibres were synthesised using 1,1,1,3,3,3-hexafluoro-2-propanol (HFIP) as the solvent (capable of solubilizing PANI-EB and PLGA), and the mixture was used to produce fibres by electrospinning. As soon as the meshes were prepared, CP was doped with HCl, changing its oxidation state from PANI-EB to PANI-ES by overnight protonation with a visible change in colour, as shown in Figure 5. The fibre alignment was analysed as a function of different proportions of PANI and PLGA, indicating that it was enhanced by increasing the amount of PLGA. It was also observed that PANI maintained conductivity in cell culture media for 100 h, after which the conductivity started to decrease because of PANI deprotonation. Culture of cardiomyocytes over the material before and after doping was positively affected by the alignment of the cells with the meshes in the doped state. ES was performed as shown in Figure 5, where 5 V cm^−1^, 1.25 Hz electrical pulses were applied (81), raising the possibility of coordinating cardiomyocyte contraction between distant cell clusters.

Transplantation of neural stem cells (NSCs) is an important tool for the recovery of injured tissue, as these cells have the ability to differentiate into neurons and other key components of the central nervous system (CNS). To avoid the random differentiation of NSCs, ES coupled with CPs as scaffold materials for cell growth had the ability to promote nerve regeneration without eliciting apoptosis due to a lack of control of exogenous factor release or high current stimulation. Xu et al. (82) developed a platform for the differentiation of NSCs based on a hydrogel (PVV-PANI) consisting of poly(2-vinyl-4,6-diamino-1,3,5-triazine) (PVDT) and 1-vinylimidazole (VI) crosslinked with poly(ethylene glycol diacrylate) coated with PANI, which was responsive to ES. The conductivity of PVV-PANI hydrogels increased by up to 5-fold when compared with PVDT without VI, indicating that the imidazole groups acted as dopants and facilitated the adsorption of PANI, which increased the electrical conductivity. The hydrogel also showed robust and elastic mechanical properties, making it a promising material for tissue engineering. The setup for cell stimulation was based on two PANI-coated ITO electrodes serving as substrates after removing the cell growth factors from the culture medium. NSCs grew for 24 h before applying a 200 Hz electric field with charge balance every other 6 h from 1 to 7 days with a biphasic pulse amplitude of 75 mV. Starting on day 5, there was a significant difference in the density of cells cultured under ES when compared with those without stimuli. A higher potential was observed to adversely affect cell growth, and the best results were obtained at 15 mV.

The effect of ES on NSC differentiation was also observed. Cells under ES showed more and longer neurites as well as more surface coverage compared with unstimulated cells. The length of the neurites also varied proportionally to the applied potential. Gene expression experiments corroborated the results, with higher values of β3-tubulin, B-FABP, and PMP22 obtained for stimulated cells, as well as genes involved in preferential differentiation to neurons and glial cells, while the value for nestin, a specific marker for NSCs, was higher in the unstimulated group.

### PEDOT-Based Materials

**Figure 6:**
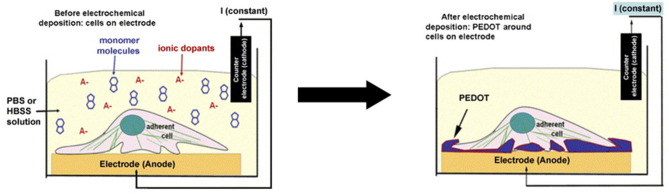
Scheme of PEDOT electrochemical deposition around the neural cell monolayer on the electrode surface. Adapted from (83) with permission from the publisher.

Another interesting study regarding material fabrication by Richardson-Burns et al. (83) studied the effects of depositing PEDOT around living neural cells. The authors studied the way the cells themselves responded to the coating and the electrochemical characteristics of the electrodes. Two different neural cell types were used: MCC and SH-SY5Y neuroblastoma-derived cell lines. Initially, cytotoxicity tests were performed with the cells using the monomer of the polymer EDOT and the stabiliser PSS^−^. The experiments showed that the cells could withstand concentrations of as high as 0.01 M EDOT and 0.02% PSS^−^ for as long as 72 h. The method of preparation of the PEDOT-coated neural cells was as follows: first, the cells were cultured over the surface of the electrodes for 24–48 h; then, the electrodes were submerged in a solution of EDOT:PSS^−^ for the subsequent electrochemical process of polymerisation. The scheme is shown below (Figure 6). This process resulted in the deposition of the polymer around the living cells. There were regions where the deposition was not present, which probably represented contact points of the cells and the electrodes where no monomer could be polymerised. This technique was subsequently implemented in the creation of cell-templated electrodes, thus creating cell-shaped cavities on their surfaces. This phenomenon was achieved by the decellularisation of the previously produced electrodes. Tests were then performed to evaluate the preference of cells in adhering to the surface. The cells seemed to have a preference for regions in which cells were present before decellularisation. However, few cells would still grow in the regions without apparent templating. PEDOT-coated cells were studied for abnormalities and defects, and despite signs of apoptosis, most of the cells were healthy after polymerisation. Apoptosis rates indeed increased, but only at 72 h after polymerisation. This finding showed that the procedure had no significant effect on nutrient transport to the cells. Increased rates of apoptosis could be attributed to subsequent permeabilisation of the cellular membrane after the electrochemical process, which was discovered by staining the cells with propidium iodide, a nucleic acid dye that is impermeable to cells with intact plasma membranes. Abnormal F-actin staining was also observable in the cells coated with PEDOT as early as 1 h after the experiment.

The study of Bolin et al. (84) presented nanofibres consisting of poly(ethyleneterphthalate) (PET) covered with PEDOT doped with tosylate. The material was used for ES of SH-SY5Y neuroblastoma cells. PET nanofibres were generated by electrospinning and covered with PEDOT by vapour phase polymerisation (VPP) of its monomer. The authors called the ES an electrochemical switch of PEDOT, for which they developed a setup that applies 3 V cm^−1^, enabling the electrochromic effect of PEDOT. At −3 V, they noticed Ca^2+^ release, and when the ES ended, Ca^2+^dropped to baseline. When the stimuli were repeated, the Ca^2+^ release was slower, and the authors correlated with voltage-operated Ca^2+^ channel desensitisation. To depolarise the membrane, KCl (50 mM) was added between the stimuli. The Ca^2+^ increase induced by the nanofibres was less steep than that induced by KCl, which could have been due to the conducting electrochemical properties of PEDOT, suggesting that the nanofibres were well-suited as electrodes for ES *in vitro*.

Another study based on PEDOT was carried out by Krukiewicz et al. (85), who studied Pt electrodes with fractal-like forms based on PEDOT/Au film for enhancing the length of neurites using mesencephalon cells. The electrode was formed by stacking layers of PEDOT:PSS (spin-coating) and Au (sputtering) over a Pt-coated glass plate, with the first layer being PEDOT:PSS and the last layer being Au. They noticed that the improvement of the layers produced electrodes with a fractal-like organisation and greater Au particles, varying from 91 nm (one layer of Au) to 702 nm (seven PEDOT/Au layers) in diameter. They discussed that the presence of water related to PEDOT:PSS creates the conditions for the gold nanoparticles to reorganise toward agglomerates and form fractal structures. The study presented different types of electrochemical characterisation, indicating that the material had a low electrical impedance of 30 ± 2 Ω at 1 Hz, charge storage capacity of 34.9 ± 2.6 mC cm^−2^, and good electrochemical stability. Other factors, such as a high signal-to-noise ratio, are important for neuronal application in ES studies.

In a more recent study, Tsai et al. (86) developed a composite electrode based on an electrospinning process with poly(ethylene oxide) (PEO), (3-glycidyloxypropyl)trimethoxysilane (GOPS) and PEDOT:PSS (87). This material showed high water resistance and retained good electrochemical performance during ES; moreover, the electrospun nanofibres possessed an efficient attraction surface, which allowed neuron adhesion and manipulation of the cell morphology. Interestingly, a spin-coated film from the same dispersion formed a contact repulsion surface that limited cell attachment. The authors suspected that this behaviour could derive from the superior nanoscale architecture in the nanofibrous material, which resembled extracellular matrixes.

**Figure 7:**
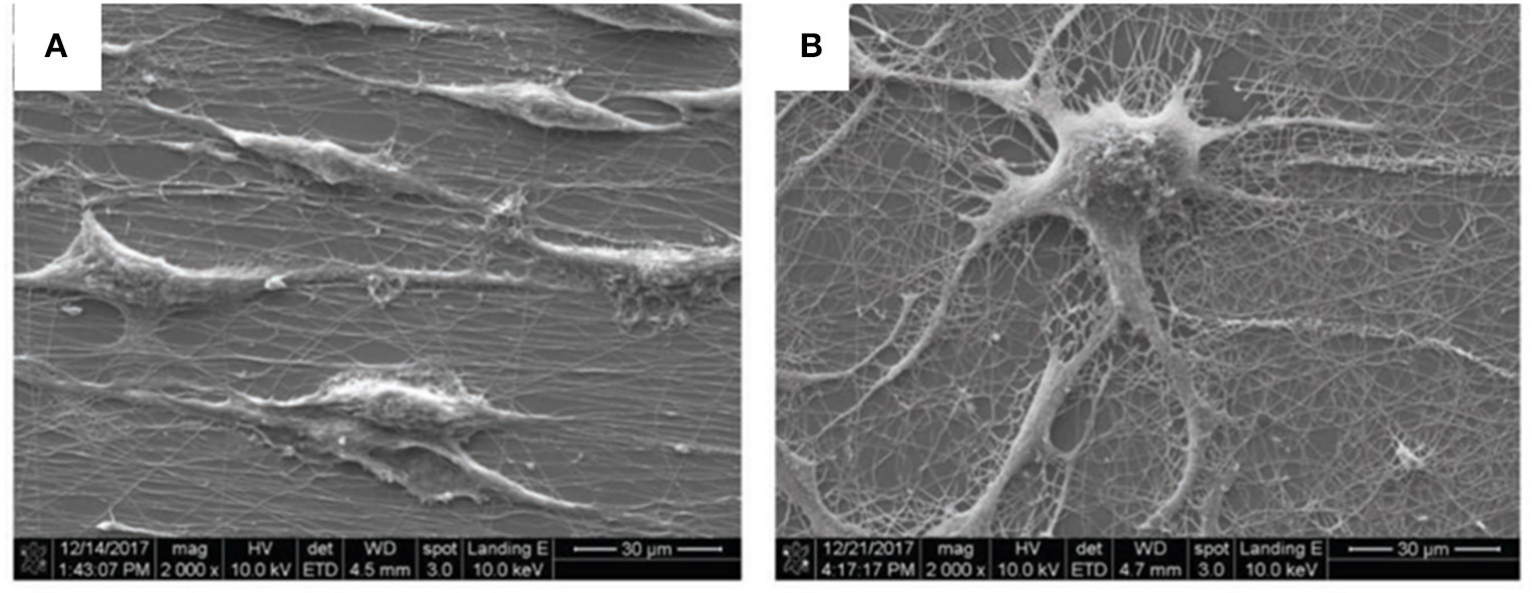
Scanning electron microscopy (SEM) images of **(A)** aligned and **(B)** random nanofibres after 5 days of culturing without ES; scale bar: 30 μm. Reproduced from Tsai et al. with permission from Wiley-VCH Verlag GmbH & Co. KGaA.

In that study, the PEO/PEDOT:PSS composite was used in the form of a thin film, with aligned or random nanofibres as a surface for differentiation of the pheochromocytoma 12 (PC12) cell line into neuron-like cells under ES. This approach is known to promote neurite growth by enhancing the MEK-ERK1/2 protein pathway (88) and by regulating protein kinase C (PKC) activity (89). The results of neurite outgrowth without ES showed that random nanofibres (Figure 7) allowed the formation of more neurites than aligned nanofibres or thin films, which showed the worst results.

**Figure 8:**
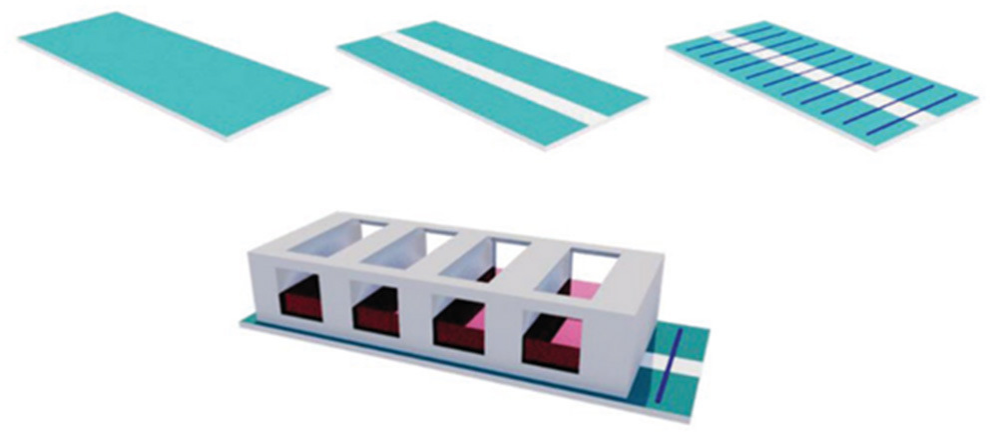
Electrical stimulation device setup with ITO substrate; ITO substrate with an etching gap; aligned nanofibers electrospun on the modified ITO substrate; aligned nanofibers electrospun on the modified ITO substrate inside a chamber slide. Reproduced from (86) with permission from Wiley-VCH Verlag GmbH & Co. KGaA.

Figure 8 shows an ES device that was mounted with PEO/PEDOT:PSS nanofibres electrospun on the surface of an ITO electrode and a chamber slide to prevent cross-contamination. PC12 cells were seeded, and after 1 day, a differentiation medium was added to ensure cell adhesion. An electrical field was provided and maintained for 5 days at an amplitude of 100 mV cm^−1^ for 1 h per day with a biphasic pulse (duration of 100 ms; interval of 100 ms).

The neurite length was increased onto the aligned nanofibres compared with the control plate surface (TCPS) and the random fibres, with the latter presenting the lowest values. The ES time was also directly proportional to the neurite length regarding both the pulse period and days of exposure. When examining gene expression, the authors observed that ES was effective in increasing the formation of RNA primers related to neural-like cells, and that longer pulses induced later stages of neural differentiation.

A compilation of the conditions used in different studies with ES, CPs and cells is systematised in Table 2.

**Table 2:** Electrical stimulation (ES) studies on different cell lines applied to conductive polymeric materials.

**Polymer**	**Cell**	**Stimulus Apparatus**	**ES**	**Scaffold**
CPSA-PANI/PLCL (90)	NIH-3T3 fibroblasts	The fibres were placed at the bottom of the cell plate among two electrodes of stainless steel	0-200 mA for 2 days	Fibres
HEC/PANI (91)	L929 cells (fibroblasts)	The cryogel was set at the bottom of a Teflon chamber with a glass window, and there weregraphite electrodes in two parallel sides of the chamber for the electric stimuli	2.5 mV/cm and 2 mA for 24 h	Cryogel
PANI (79)	Human osteosarcoma (HOS)	A polystyrene ring was set over the interdigitated electrodes (IDEs) to make a well for the cell culture, the ends of the IDEs remained outside of the well and were connected to wires using a silver glue	1 kHz, 0-1600 mV	Films on interdigitated electrodes (IDEs)
PANI (92)	Human mesenchymal stem cells (hMSCs)	PANI films were placed at the centre of the tissue culture plates and at the opposite ends of the plate two stainless steel electrodes	1 mV/cm^−2^ V/cm for 2 min per day	Film
PANI/Coll/sHya (93)	Human mesenchymal stem cells (hMSCs)	The material was placed at the bottom of the cell plate, the cell media used in the cell culture chamber was cycled by a silicone tube exposed to the electric stimuli	7 ms rectangular pulses, 3.6 mV/cm, 10 Hz	Artificial Extracellular Matrix (aECM)
PANI/PLGA (80)	Cardiomyocyte	The nanofibre mesh was set at the bottom of a glass well, and two silver wires are attached at the walls to promote the electric stimuli	1.25 Hz, 5 V/cm	Fibres
PANI-PCL (94)	Human umbilical vein endothelial cells (HUVECs)	The fibre film was set between two electrodes for electric stimuli	200, 300 and 400 mV/cm in 30 min per day for 4 days	Film
PANI-Pt (95)	Rat retinas	The PANI-coated Pt electrode acted as the anode, and a gold electrode acted as the cathode	100-μA, 0.8-ms pulse width and 1-s repeat interval stimulating a biphasic rectangular current pulse	Neural probe
PCL/PPy and PCL-PP/HEP (73)	Haemocompatibility	The mesh was set at the bottom of the well, and two platinum wires were attached to opposite ends of the mesh using a holder to prevent the wires from remaining in contact with the cell media	10 μA, 100 Hz AC for 2 h	Fibres
PCL-PPy-PSS (96)	Human mesenchymal stem cells	The fibres were set over a glass plate attached with cooper tape at the opposite ends for electric stimuli, and polycarbonate was placed over the fibres for the cell culture	10 mV mm^−1^ for 8 h	Fibres
PEDOT:PSS (97)	Neural stem cells (NSC)	An adhesive silicone ring was placed over the material, two gold parallel strips were deposited at the edges of the ring and platinum wires were connected to those strips for electric stimuli	100-Hz pulsed DC electrical stimulation, 1 V with 10-ms pulses over 12 h per day	Films
PEDOT:PSS (98)	Neurons	A grade of adhesive silicon was used to attach the MEA device and build the cell culture well	30 pulses at a frequency of 1 Hz	Macroporous
PEDOT:PSS or IrOx or (Ir-Ti)Ox or Au (99)	Spinal *X. laevis* neurons	The material was set at the bottom of a modified chamber with a glass cover, making a channel for the cells and the cell media; at the opposite ends of the material were two wells with cell media connected by agar bridges to two baths of Steinberg's solution and Ag/AgCl electrodes for the electric stimuli	50, 100, or 150 mV/mm for 3 h	Films
PEDOT:PSS with LCGO AND PU (PUHC) (100)	Neural stem cells	The hydrogel was set at the bottom of the well where the cell culture chamber had a bottom of gold mylar, and the mylar was connected to platinum wires for the electric stimuli	The stimulation paradigm was ±0.25 mA cm^−2^ using a biphasic waveform of 100-μs pulses with a 20-μs interphase at 250 Hz over 8 h per day for 3 days	Hydrogel
PLLA/PANI (101)	Neural stem cells (NSCs)	A platinum electrode was attached on one side and a silver electrode on the other at the end of the fibres	1.5 V (100 mV/mm) for 1 h	Fibres
PLLA/PPy/HE (65)	Osteoblast (Saos-2)	The membrane was placed in the well, and the edges were connected to a source of electrical stimulus	200 mV/mm	Membrane
PPy (64)	Bone marrow stromal cells (BMSC)	PPy acted as the anode, and lengthways to the well, a gold wire acted as the cathode; the system also contained a silver wire as a quasi-reference electrode	20 V/m for 1 h	Film over ITO
PPy (102)	Schwann cell	The material film fixed with PMDS was places in a hole at the centre of a Petri dish, and cooper tape was used for the electric contact with the film for the electric stimuli	0.1, 0.5, 1.0 V for 2 h	Film over ITO
PPy/pTS/NT3 (103)	Spinal ganglion neurons	The film was placed at the bottom of the cell plate, and gold electrodes were placed at the walls of the wells	Charged-balanced biphasic current pulses at 250 Hz were applied for 1 h. The waveform had a ±1 mA current amplitude, 100-ms pulse width, 20-μs open-circuit interphase gap and 3.78-ms short-circuit phase between pulses	Film over gold
PPy-HA and PPy-CS (104)	Adipose stem cells (hASCs)	A gold electrode covered with the PPy film was placed at the bottom of the well, and gold electrodes were submerged in the cell media for the electric stimuli	Biphasic electric current (BEC)of ± 0.2 V amplitude, 2.5-ms pulse width and 100-Hz pulse repetition frequency	Film
PPy-PLA and PPy-PCL (73)	Dorsal root ganglia	Silver electrodes were connected to the opposite edges of the fibres for the electric stimuli	10 V	Fibres
PPy-PLGA (77)	Retinal ganglion cells	The nanofibres were set in a Petri dish, and the opposite ends of the fibres were connected to platinum wires	−0.1 to −1 V/cm over 1 h per day for 3 days	Fibres
PVV-PANI (82)	Neural stem cells (NSCs)	The hydrogel sheet was deposited on an ITO electrode, and two electrodes were used for the electric stimuli in the cell culture homemade chamber	Biphasic electrical field with 200 Hz, amplitude of the biphasic pulse fixed at 75 mV	Hydrogel
SPAN (105)	Bone marrow stromal cells (BMSC) and pre-osteoblast cells (MC3T3-E1)	A polystyrene ring was set over the interdigitated electrodes (IDEs), making a well for the cell culture, and the ends of the IDEs remainedoutside of the well and were connected to wires using a silver glue	1 kHz, 500 mV	Films on interdigitated electrodes (IDEs)
VPP:PEDOT (PET/PEDOT:pTS (84)	SH-SY5Y neuroblastoma	Two fibre electrodes were layered at the bottom of the cell plate with a gap between them. The fibre electrodes were in contact with a silver tape for the electric stimuli	−3.0 V	Fibres

## Discussion

To compare different setups with CPs and ES, we decided to look at studies with PC12 cells, and their conditions are summarised in Table 3. Within these studies, Zou et al. (114) prepared a material of PPy-PDLLA and applied electric fields of 100, 200, 400, and 800 mV cm^−1^ to PC12 cells for 4 h. The material was made by electrospinning, and they could control fibre formation, making films of both random and aligned fibres. They noticed that the material organisation without ES was able to orient the cells, and this orientation also increased the neurite length from 65 μm in the random fibre film to 114 μm on average for the aligned fibres. When an electric field was applied, the effect on cells over the aligned fibres was enhanced, especially at an applied potential of 200 mV cm^−1^. ES also promoted an enhancement of cell differentiation and cell alignment over the fibres.

**Table 3:** Various conditions used in various studies with ES of PC12 cells.

**Polymer**	**Stimulus apparatus**	**ES**	**Scaffold**
PANI on ITO (106)	The ITO covered with a PANI film was set at the bottom of the cell culture plate, and two wires were set in contact with the well for the electric stimuli	100-μA amplitude, 0.8-ms pulse width, and 1-s repeat interval stimulating the biphasic rectangular current pulse for 1, 2, and 4 h	Film
PCLF-PPy (107)	The membrane was placed at the bottom of the well, and a silicon tube with two platinum wires was placed in the well to make contact with the membrane scaffold without exposing the wires to the culture medium	10 μA or 20 Hz for 1 h/d for 2 days	Membrane
PEDOT:PSS (108)	The opposite ends of the patterned nanoparticles arrays were painted with silver epoxy electrodes for the electric stimuli	Monophasic pulsed current at 250 Hz with a 2-ms pulse width and an amplitude of 1 mA for 2 h	Patterned multifunctional PEDOT:PSS nanoparticle arrays
PEO/PEDOT:PSS (86)	The cell chamber was made over an ITO electrode coated with the material, and the electrical stimuli were applied to the ITO	Biphasic square wave (100 mV cm^−1^ electric field; duration of 100 ms; interval of 100 ms) in 1 h/d for 5 days	Fibres
PLAAP (109)	The membrane was placed at the bottom of the cell plate, and two platinum electrodes were placed in contact with the lengthwise boundaries of the membrane	1 Hz, 0.1 V in 1 h/d for 4 days	Film
PPy/PDLLA (110)	The conduits were placed between a PDMS film and a PMDS well-attached to two silver wire electrodes	100 mV for 2 h	Conduit
PPy (24)	PPy acted as the anode, and lengthways to the well a gold wire acted as the cathode; the system also had a silver wire as a quasi-reference electrode	100 mV for 2 h	Film over ITO
PPy-coated PLGA (111)	The fibres were placed between a PDMS film and a PMDS well, which were attached to two silver wire electrodes	100 mV/cm or 10 mV/cm for 2 h	Fibres
PPy-CS and PPy-CS-Col (112)	The material was set at the bottom of the cubic chamber, and on two opposite sides of the well were PVDF sheets to link the media to the other two wells, whichwere connected to stainless steel meshes for the power supplier	±1-m A pulse duration of 0.1 ms, with a steady state interval of 3.8 ms for 144 h	Film
PPy-NGF (113)	PPy acted as the anode, and lengthways to the well a gold wire acted as the cathode; the system also had a silver wire as a quasi-reference electrode	100 mV for 2 h	Films
PPy-PLLA (114)	The fibres were placed between a PDMS film and a PMDS well-attached to two gold wire electrodes	100, 200, 400, and 800 mV/cm for 4 h	Fibres
PS/PANI (115)	The fibres were set at the bottom of the well with one end of the mesh connected with a platinum and the other with a silver electrode	100 mV/cm in 1 h/d for 5 days	Fibres

**Figure 9:**
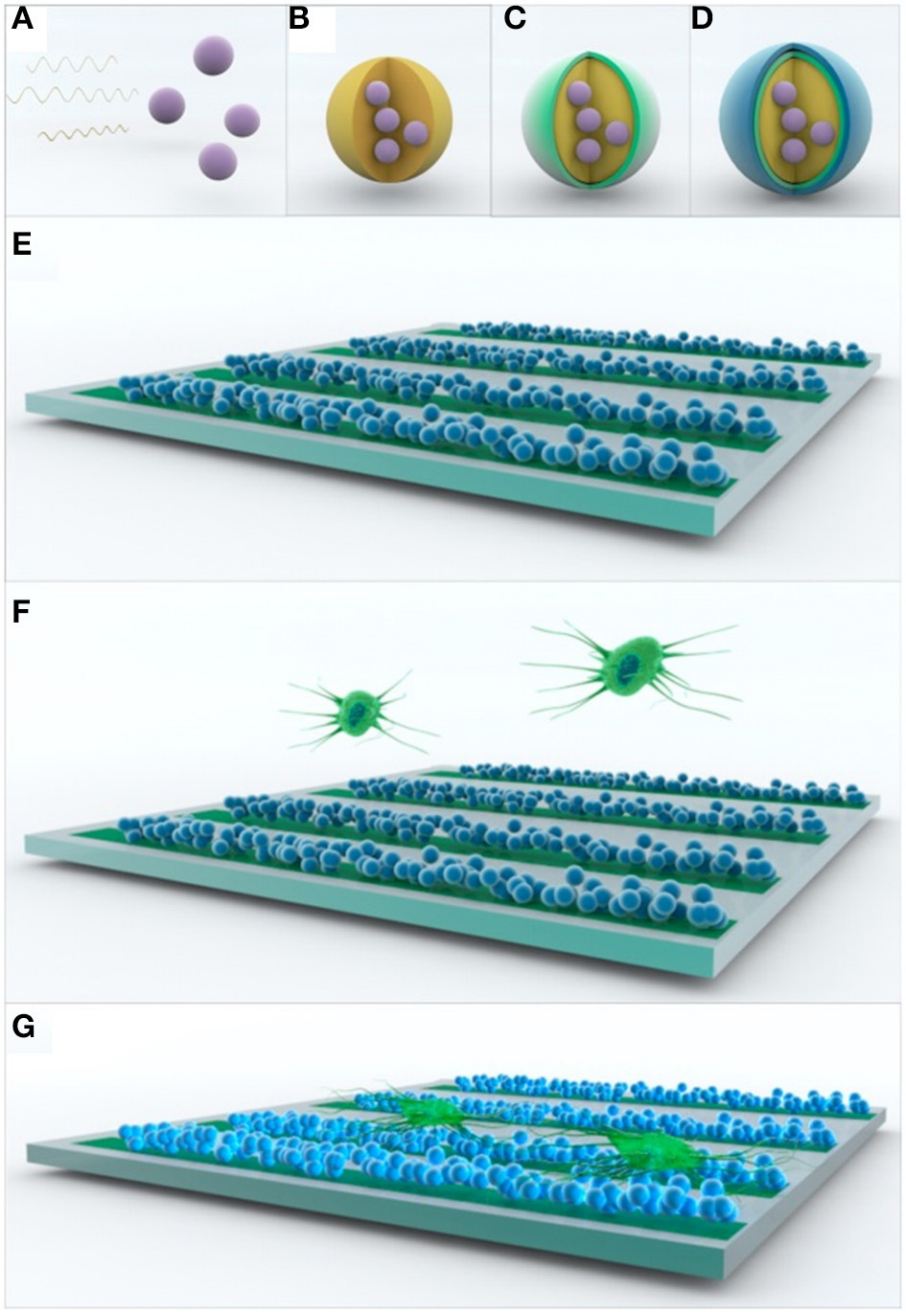
Scheme of the assembly of multifunctional PEDOT:PSS nanoparticle arrays for ES. **(A–D)** Multilayer formation of PEDOT:PSS nanoparticles via non-spontaneous emulsification. **(E–G)** Patterning of the multilayered PEDOT:PSS nanoparticles for ES of PC12 cells. Reproduced from (108) with permission from the publisher.

Ho et al. (108) also noticed that a patterned scaffold based on PEDOT:PSS nanoparticles structured in a conduit pattern over an ITO substrate could enhance the adhesion of PC12 cells (Figure 9). By applying pulses at 250 Hz for 2 ms with an amplitude of 1 mA, it was noticed that the cells improved the dendritic network over the aligned fibre scaffold.

These studies showed that the alignment of the fibres in the scaffold influenced cellular growth, and, therefore, on the device setup for the ES experiments, the fibres were connected in a manner to allow the flux of electrons to follow the fibres in the material.

**Figure 10:**
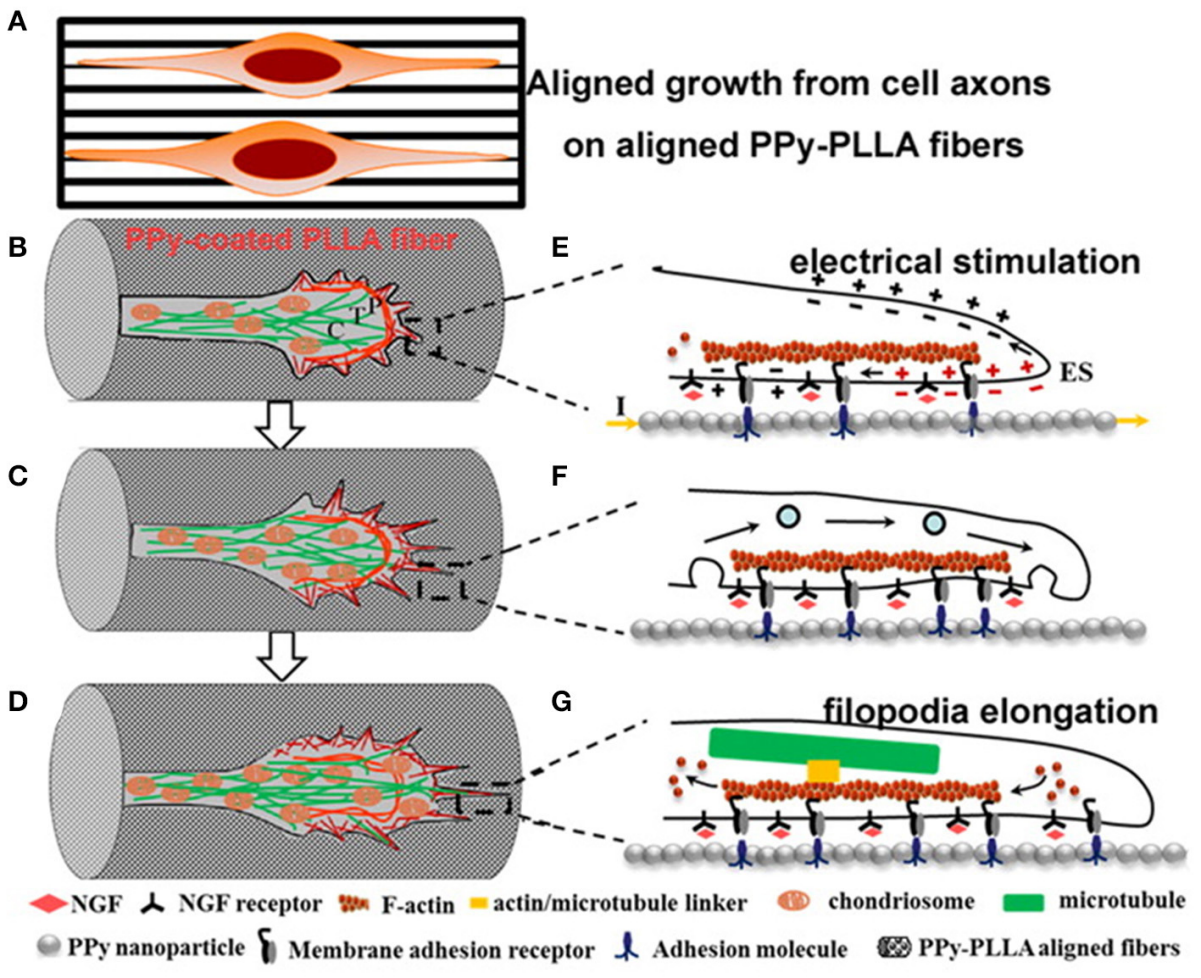
Scheme of axon elongation from PC12 cells on aligned fibres **(A)** after differentiation, **(B–D)** change in growth cone, and **(E–G)** inner change in filopodia during elongation. Reproduced from (114) with permission from the publisher.

To understand the effect of an organised pattern, Zou et al. (114) proposed a mechanism that correlates the CP surface during ES with the neural growth cone, and they showed that the enhancement of charge in a rough PPy-PDLLA fibre surface could trigger processes of molecule adhesion and neural growth cones in different domains (peripheral, P, transition zone, T, and central domain, C), creating guidance for neuron outgrowth along the PPy-PDLLA fibres (Figure 10).

Together with the material scaffold, ES conditions are also important to modulate cell growth and differentiation. Moroder et al. (107) proposed a study on two different ES routines over polycaprolactone fumarate–PPy (PCLF–PPy) scaffolds to stimulate PC12 cells. The setups promoted 10 μA current stimuli over the membranes, one with a constant direct current applied for 1 h per day for 2 days, and the other with a pulse frequency of 20 Hz. They noticed that the membrane of PCFL-PPy treated with naphthalene sulphonic acid (NSA) and with an ES of 20 Hz frequency resulted in an increase in the neurite number per cell in comparison to the constant one, but both ES protocols promoted enhancement of the neurite length per cell over the non-stimulated culture. Taking that study into account, the choice of ES conditions needs to be tested with the cells and the material so that the difference in material conductivity can change the current density felt by the cells and hinder the current density measurement given that the geometric areas of fibres or membranes do not represent the total area of the material (116).

In summary, for PC12 cells, an ES of at least 10 mV over a time range of 2–5 h in total was sufficient to promote cell outgrowth (24, 110, 111, 113–115, 117), This finding indicates that a continuous stimulus for a defined period per day can promote cell growth without damaging the cells or the electrode.

When comparing studies related to neurons, Rajnicek et al. (98) studied the effects of an applied electric field over amphibian neurons growing on transparent films of diverse conductive materials, such as bare metals, polymers, and semiconducting oxides. The applied electric field was not direct; hence, it was applied by connecting electrodes to the solution in which the materials were immersed, generating a dipole wirelessly within them. The electric field was from a DC power supply connected to two Ag/AgCl electrodes in baths of Steinberg's solution. Electrical contact with the cell cultures was made through two 2%_w/v_ agar bridges, with one end of each bridge in the electrode bath and the other in the pools of culture medium at each end of the channel. The medium was constrained by dams of silicone. The electric field was set by measuring the voltage across the chamber length to yield 50, 100, or 150 mV mm^−1^.

To date, studies conducted on mammalian cells have been limited and have generally focused on hippocampal neurons in the CNS in the absence of glia. Embryonic rat hippocampal neurons were submitted to constant DC fields of 28–219 mV mm^−1^ for 24 h and responded by extending the outgrowth perpendicular to the applied ES with less growth in the cathode direction (118). A higher non-physiological applied ES (>580 mV mm^−1^) caused embryonic rat hippocampal neuronal growth cones to turn in the direction of the cathode (119). A recent study also showed that ES increased the survival rates of retinal ganglion cells after the optical nerve had been damaged (120).

The topography and chemistry of the growth substrate also influence the ES neuronal response. As an example, neurites extending from embryonic frog spinal neurons showed not only electrical cues but also guidance cues, and the neurites extended orthogonally to the applied electrical field (121).

These findings explain how many factors play key roles in *in vitro* ES studies. As an example, astrocytes are the most abundant glia in the CNS and have various functions, such as in the nutrient supply, neurotransmission, and structural support. They are known to orient themselves perpendicular to a highly exogenous non-physiological electrical field (122). This behaviour is instructive for neurons, and when controlled by an exogenous stimulus, they can provide benefits of assisting in neuronal injury repair. Another example is oligodendrocytes, which are an important myelinating agent in CNS glia. Experiments have shown that controlled applied electrical fields exhibited higher rates of neuronal survival than control fields (123). These experiments clearly showed that glia of the CNS responded differently following ES. The mechanisms to explain such responses need to be further investigated until an improved understanding is achieved.

A major concern when applying *in vitro* ES of CP-based materials is the effect on water electrolysis and consequently on the medium pH, which can affect cell viability (51, 88). Cell culture with ES can also promote electrode corrosion (42), and, thus, some studies have combined biodegradable materials with CPs to avoid generating toxic species in the media (124–126). Biphasic stimuli represent a process that changes the current direction during pulses, a procedure that is interesting for reducing electrode damage by Faradaic reactions at the electrode surface (42, 44).

Moreover, it is important to maintain the current density within a range without causing cell damage, and the current density is dependent not only on the ES but also on material conductivity, number of cells, and distance between the electrodes (127). Changing these parameters could change the current density through the cells. Hence, the material properties together with ES generate responses in the molecules of the cell membrane, leading to accelerated growth and differentiation processes, which is a beneficial and key aspect of tissue regeneration.

## Conclusion

*In vitro* ES using CPs needs to consider the design of the material as well as the type of cell in relation to the stimulus control. In the case of different cells, the ES duration may vary: for cardiomyocytes, it is interesting to apply a short-period stimulus to monitor contraction of the cells during stimulation, while for neuronal cells, longer ES periods are necessary to monitor the cells after each stimulus to confirm the cell viability. Biphasic stimulation is also an important and viable alternative to reduce cell and electrode damage during long ES periods. The addition of growth factors to the cell media or when immobilised on the CP surface could also affect the necessary ES duration.

Electrical stimulation can be performed in various devices, some of which make use of the CP itself as a working electrode, allowing the stimulus to be applied directly on the material or as thin films deposited on conductive supports, such as ITO and gold mylar. Other possible designs make use of a salt bridge that generates a flux of electrons in the culture medium. Overall, the choice of devices *in vitro* must acknowledge that the material should generate a cell stimulus without eliciting any toxic effects. Another important aspect to consider is to construct the device with the material synthesis already envisioning the *in vivo* application.

In summary, CPs and ES are important tools to study cell growth for *in vitro* tissue regeneration and are relevant to understanding and tailoring future applications of these materials in *in vivo* devices.

## Author Contributions

All the authors contributed to the writing and discussion of the study.

## Conflict of Interest

The authors declare that the research was conducted in the absence of any commercial or financial relationships that could be construed as a potential conflict of interest.

## Publisher's Note

All claims expressed in this article are solely those of the authors and do not necessarily represent those of their affiliated organizations, or those of the publisher, the editors and the reviewers. Any product that may be evaluated in this article, or claim that may be made by its manufacturer, is not guaranteed or endorsed by the publisher.
